# Prevalence, Genetic Diversity, and Risk Factors of *Cryptosporidium* spp. in HIV/AIDS Patients: An Updated Systematic Review and Meta‐Analysis (2017–2025)

**DOI:** 10.1155/cjid/2740716

**Published:** 2026-06-12

**Authors:** Farzad Mahdavi, Mohammad Reza Mohammadi, Shayan Heidari, Roumina Norouzi, Ali Pouryousef, Kambiz Karimi, Asma Mousivand, Mahtab Mehboodi, Ali Asghari, Hassan Nourmohammadi

**Affiliations:** ^1^ Department of Medical Parasitology and Mycology, School of Medicine, Zanjan University of Medical Sciences, Zanjan, Iran, zums.ac.ir; ^2^ Department of Bacteriology, Faculty of Medical Sciences, Tarbiat Modares University, Tehran, Iran, modares.ac.ir; ^3^ Medical Faculty, University of Belgrade, Belgrade, 11000, Serbia, bg.ac.rs; ^4^ Leishmaniasis Research Center, Sabzevar University of Medical Sciences, Sabzevar, Iran, medsab.ac.ir; ^5^ Department of Parasitology and Mycology, Faculty of Medicine, Kurdistan University of Medical Sciences, Sanandaj, Iran, muk.ac.ir; ^6^ Department of Pathobiology, School of Veterinary Medicine, Shiraz University, Shiraz, Iran, shirazu.ac.ir; ^7^ Department of Microbiology and Virology, School of Medicine, Kerman University of Medical Sciences, Kerman, Iran, kmu.ac.ir; ^8^ Medical Microbiology Research Center, Qazvin University of Medical Sciences, Qazvin, Iran, qums.ac.ir; ^9^ Zoonotic Diseases Research Center, Ilam University of Medical Sciences, Ilam, Iran, medilam.ac.ir; ^10^ Department of Hematology & Oncology, School of Medicine, Ilam University of Medical Sciences, Ilam, Iran, medilam.ac.ir

**Keywords:** AIDS, *Cryptosporidium*, HIV, prevalence, species, systematic review

## Abstract

**Background:**

*Cryptosporidium* spp. is a major opportunistic pathogens in HIV/AIDS patients, contributing substantially to morbidity and mortality worldwide. Despite advances in HIV/AIDS management, the global burden, genetic diversity, and risk factors of cryptosporidiosis in this high‐risk group remain incompletely understood.

**Methods:**

We conducted a systematic review and meta‐analysis of cross‐sectional and case–control studies published between January 1, 2017, and June 10, 2025, in accordance with PRISMA 2020 guidelines. Literature searches were performed in PubMed, Scopus, Embase, and Web of Science, supplemented by Google Scholar. Eligible studies reported the prevalence of *Cryptosporidium* spp. in HIV/AIDS patients, with or without comparison groups, using microscopy, serology, or molecular methods. Data extraction was performed independently by two reviewers, and study quality was assessed using the JBI checklist. Random‐effects models were applied in Comprehensive Meta‐Analysis software, with subgroup analyses and meta‐regression conducted to explore prevalence and heterogeneity.

**Results:**

A total of 89 studies were included. The pooled prevalence of *Cryptosporidium* spp. among HIV/AIDS patients was 9.5% (95% CI: 7.6–11.8). Based on 17 case–control studies, HIV‐positive individuals had a significantly higher risk of infection compared to HIV‐negative controls (OR: 3.5, 95% CI: 2.1–5.9). Subgroup analyses revealed higher pooled prevalence in smaller studies, medium‐HDI and lower‐middle‐income countries, and African and Central American settings. Pooled prevalence did not differ significantly across diagnostic methods. Among HIV/AIDS patients positive for *Cryptosporidium* spp., 39.8% were aged ≤ 30 years and 64.7% were > 30 years. Females represented 54.5% of positives, slightly higher than males at 45.8%. Most infected patients had CD4 counts ≤ 200 cells/μL (62.5%), compared to 43.2% with counts > 200. Diarrhea was present in 78.4% of positive cases, versus 43.8% among those without infection. Meta‐regression confirmed a significant inverse association between sample size and prevalence (*β* = −0.0019, *p* = 0.003). Molecular data identified diverse species and subtypes, with *C. hominis* and *C. parvum* predominating but zoonotic species such as *C. meleagridis*, *C. felis*, *C. viatorum*, *C. canis*, *C. suis*, *C. andersoni*, and *C. cuniculus* also reported. Sensitivity analyses showed no single study significantly influenced the pooled prevalence. Funnel plot asymmetry and Egger’s test indicated publication bias.

**Conclusions:**

This updated synthesis demonstrates that cryptosporidiosis remains a substantial health burden in HIV/AIDS patients, with notable genetic diversity reflecting both anthroponotic and zoonotic transmission routes. The strong association with immunosuppression, as evidenced by low CD4 counts and diarrheal symptoms, underscores its clinical relevance. These findings highlight the urgent need for improved surveillance, molecular epidemiology, and preventive interventions to mitigate the impact of *Cryptosporidium* spp. in vulnerable populations.

## 1. Introduction

*Cryptosporidium* species are protozoan parasites belonging to the phylum Apicomplexa that invade the epithelial lining of the gastrointestinal tract in a wide array of vertebrate hosts, including humans [1]. The resulting disease, cryptosporidiosis, is an enteric infection most commonly manifested by watery diarrhea [2]. The clinical course can range from acute and short‐lived to persistent or chronic, largely depending on the host’s immune competence [3]. Globally, cryptosporidiosis represents a significant contributor to diarrheal disease, particularly in children and in immunocompromised individuals, and remains one of the leading waterborne parasitic infections with considerable public health implications [4, 5].

Transmission occurs primarily through the fecal‐oral route following ingestion of environmentally resistant oocysts that are shed in high numbers in the feces of infected hosts [6]. Major sources of exposure include contaminated drinking water, recreational water, and food, while direct human‐to‐human and zoonotic transmission routes are also well established [7, 8]. In immunocompetent individuals, the infection is often self‐limiting and characterized by diarrhea, abdominal cramps, nausea, and mild fever. In contrast, patients with compromised immune systems, particularly those with advanced human immunodeficiency virus (HIV)/acquired immunodeficiency syndrome (AIDS), frequently experience chronic, severe, and even life‐threatening disease associated with dehydration, malnutrition, and wasting [9, 10].

*Cryptosporidium* spp. is a well‐recognized zoonotic parasite with a remarkable ability to infect a wide range of hosts, including mammals, birds, and reptiles [11]. Molecular epidemiological studies have consistently shown that *C. hominis* (primarily transmitted between humans) and *C. parvum* (with both human and animal reservoirs) are the most prevalent species causing human infection worldwide. Nonetheless, a growing number of additional species and genotypes with zoonotic potential have been identified in humans, including *C. meleagridis*, *C. canis*, *C. felis*, *C. ubiquitum*, *C. cuniculus*, *C. viatorum*, *C. muris*, *C. andersoni*, *C. erinacei*, *C. tyzzeri*, *C. bovis*, *C. suis*, *C. scrofarum*, *C. occultus*, *C. xiaoi*, *C. fayeri*, *C. ditrichi*, *C. mortiferum*, and several host‐adapted genotypes such as those from mink, skunk, and horse. This diversity reflects the parasite’s extraordinary host adaptability and underscores the complexity of cross‐species transmission [12–14].

Despite advances in antiretroviral therapy (ART) and improvements in HIV care, cryptosporidiosis remains an important opportunistic infection in patients with impaired immunity [15]. It continues to pose a major clinical challenge in HIV/AIDS patients and highlights the need for up‐to‐date evidence on its prevalence, genetic diversity, and associated risk factors. A prior systematic review and meta‐analysis published in 2020 synthesized available data but was restricted to studies conducted up to 2017 [16]. Since then, numerous new epidemiological and molecular studies have been published, providing an expanded evidence base that has yet to be comprehensively analyzed.

Accordingly, the present study was undertaken with the following objectives: (1) to provide an updated global estimate of the prevalence of *Cryptosporidium* spp. among HIV/AIDS patients from January 1, 2017, to June 10, 2025; (2) to characterize the species and genotypic/subtypic diversity of *Cryptosporidium* spp. in this population; and (3) to assess potential risk factors through various statistical analyses. By addressing these aims, this review seeks to bridge existing knowledge gaps and supply a contemporary evidence base to inform clinical management and public health strategies targeting cryptosporidiosis in immunocompromised populations.

## 2. Methods

### 2.1. Study Design and Reporting

This study was designed as a systematic review and meta‐analysis and was conducted and reported in accordance with the Preferred Reporting Items for Systematic Reviews and Meta‐Analyses (PRISMA) 2020 guidelines [17]. A protocol was developed a priori, specifying the objectives, eligibility criteria, search strategy, data extraction process, quality assessment, and statistical methods.

### 2.2. Search Strategy

A comprehensive literature search was carried out in PubMed, Scopus, Embase, and Web of Science for studies published between January 1, 2017, and June 10, 2025. The search strategy combined Medical Subject Headings (MeSH) and free‐text terms related to *Cryptosporidium*, cryptosporidiosis, intestinal parasites, immunocompromised patients, HIV, AIDS, prevalence, epidemiology, species, genotype, subtype, genetic diversity, and genotyping. The full search syntax was adapted for each database. To minimize publication bias and avoid the possible missing of some studies, we also searched Google Scholar and manually screened reference lists of relevant articles and reviews. No language restrictions were applied, and non‐English papers were included if sufficient data could be extracted.

### 2.3. Eligibility Criteria

Studies were eligible if they met the following criteria: (1) full‐text availability; (2) reported the prevalence of *Cryptosporidium* spp. in HIV/AIDS patients, with or without a non‐HIV control group; (3) diagnosis based on microscopic, serological, or molecular methods; and (4) reported both total sample size and number of positive cases, allowing prevalence to be calculated. Exclusion criteria included studies with insufficient or non‐extractable data and unavailability of the full text, unclear or incomplete information, as well as reviews, editorials, case reports, letters, and conference abstracts lacking complete data. Studies conducted on immunocompromised patients other than those with HIV/AIDS and studies restricted exclusively to healthy individuals were also excluded.

### 2.4. Data Extraction

Two independent reviewers with expertise in parasitology extracted data using a standardized prepiloted checklist. Discrepancies were resolved through discussion or consultation with a third reviewer. Extracted variables included the first author’s name, year of publication and year of implementation, study design (cross‐sectional or case–control), total sample size, and number of positive cases, prevalence (%) of *Cryptosporidium* spp. in HIV‐positive and HIV‐negative groups (if available), geographical location and country income level, Human Development Index (HDI) category, diagnostic methods applied (microscopy, serology, and molecular), and *Cryptosporidium* species and genotypes/subtypes identified.

### 2.5. Quality Assessment

The quality of included studies was appraised independently by two reviewers using the Joanna Briggs Institute (JBI) critical appraisal checklists: the 8‐item tool for cross‐sectional studies and the 10‐item tool for case–control studies. Studies scoring 4–6.5 points were classified as moderate quality, and those scoring ≥ 7 points as high quality [18]. Discrepancies were resolved by consensus.

### 2.6. Statistical Analysis

All statistical analyses were performed using Comprehensive Meta‐Analysis (CMA) software Version 3. The pooled prevalence of *Cryptosporidium* spp. among HIV/AIDS patients was calculated using a random‐effects model with 95% confidence intervals (CIs) [19]. Heterogeneity was assessed using the *I* ^2^ statistic, with *I* ^2^ > 75% considered substantial heterogeneity. Odds ratios (ORs) were calculated from case–control studies comparing HIV‐positive patients with HIV‐negative controls. Subgroup analyses were performed according to year of publication, sample size, continent, country, country income level, HDI, diagnostic method, and study design. Sensitivity analyses were conducted by sequential omission of individual studies, and meta‐regression was used to explore the effect of continuous variables (sample size, publication year, HDI, and country income level) on pooled prevalence. Publication bias was evaluated using funnel plots and Egger’s regression test. A two‐tailed *p*‐value < 0.05 was considered statistically significant.

## 3. Results

### 3.1. Study Selection

**FIGURE 1:**
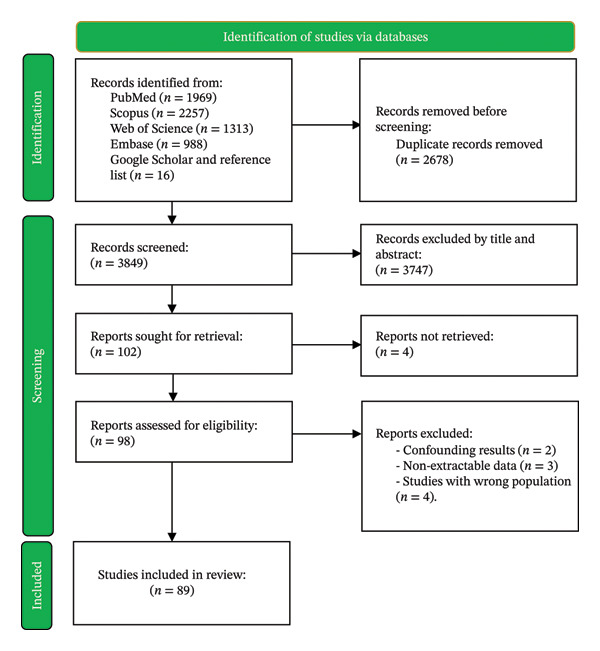
The PRISMA 2020 flow diagram depicting the process of included studies in the present systematic review.

The database search initially yielded 6527 records, with an additional 16 identified through Google Scholar and reference list screening. After removal of duplicates (*n* = 2678) and screening of titles and abstracts (*n* = 3747 excluded), 98 full‐text articles were assessed for eligibility. Ultimately, 89 studies met the inclusion criteria and were included in the meta‐analysis [20–30], [31–40], [41–50], [51–60], [61–70], [71–80], [81–90], [91–100], and [101–108]. A PRISMA 2020 flow diagram illustrates the reporting and study selection process (Figure 1).

### 3.2. Characteristics of Included Studies

The included studies comprised 72 cross‐sectional and 17 case–control designs, published between 2017 and 2025. A total of 20,065 HIV‐positive patients and 4564 HIV‐negative controls were evaluated. Studies were geographically distributed in 28 countries across four continents, with the highest representation from Africa (58 studies), followed by Asia (26 studies), South America (three studies), and Central America (two studies). Diagnostic approaches included microscopy (61 studies), serology (four studies), and molecular methods (24 studies). Of 24 molecular studies, 14 reported *Cryptosporidium* spp. distribution and/or subtypes in HIV/AIDS patients (Table 1).

**TABLE 1:** The main data from 89 studies (72 cross‐sectional and 17 case–control) on the prevalence and genetic diversity of *Cryptosporidium* spp. in HIV/AIDS patients and healthy controls.

Author, year	Time‐tested	Country	Cases		Controls		Study type	Methods	*Cryptosporidium* species (genotype/subtype) in HIV/AIDS patients
			Total no.	Prevalence (%)	Total no.	Prevalence (%)			
Anejo‐Okopi, 2017	2015–2016	Nigeria	296	4.7	—^b^	—^b^	CS^c^	MIC^e^	—
Zida, 2017	2013–2014	Burkina Faso	131	21.4	—	—	CS	MIC	—
Uysal, 2017	UC^a^	Turkey	115	2.6	—	—	CS	MOL^f^	—
Ukwah, 2017	UC	Nigeria	251	6.8	143	5.6	CC^d^	MOL	*C. parvum* (IIcA5G3b, IIeA10G1, IIcA5G3k), *C. hominis* (IaA27R4, IeA11G3T3, IaA18R3, IbA10G2, IaA29R3), *C. felis*, and *C. viatorum*
Tay, 2017	2011	Ghana	341	2.1	331	0	CC	MIC	—
Swathirajan, 2017	2007–2014	India	829	1.2	—	—	CS	MIC	—
Sharma, 2017	2016–2017	Nepal	193	2.1	111	0	CC	MIC	*C. parvum*
Sandhya, 2017	2014–2016	India	110	77.3	—	—	CS	MIC	—
Olopade, 2017	2013	Nigeria	226	4.4	—	—	CS	MIC	—
Obateru, 2017	2010–2011	Nigeria	238	55	238	16.8	CC	MIC	—
Nsagha, 2017	2014	Cameroon	300	44	—	—	CS	MIC	*C. parvum*
Liyasu, 2017	UC	Nigeria	150	39.3	50	38	CC	MIC	*C. parvum*
Cerveja, 2017	2015–2016	Mozambique	371	0.3	146	0	CC	MIC	—
Eshetu, 2017	2016	Ethiopia	223	3.1	—	—	CS	MIC	*C. parvum*
Gedle, 2017	2015–2016	Ethiopia	323	5.9	—	—	CS	MIC	—
Zorbozan, 2018	UC	Turkey	65	21.5	—	—	CS	MIC	—
Akgul, 2018	2015–2016	Turkey	90	3.3	—	—	CS	MOL	—
Alemu, 2018	2016	Ethiopia	220	8.6	—	—	CS	MIC	—
Amoo, 2018	UC	Nigeria	231	22.5	—	—	CS	MIC	—
Barcelos, 2018	2015–2016	Brazil	90	1.1	—	—	CS	MIC	—
Casmo, 2018	2011–2013	Mozambique	83	7.2	25	12	CC	MOL	*C. hominis* (IaA23R3) and *C. parvum* (IIcA5G3d)
Fallahi, 2018	2016	Iran	120	10	—	—	CS	MOL	—
Gebretsadik, 2018	2016	Ethiopia	223	1.4	—	—	CS	MIC	—
Ghafari, 2018	2014–2015	Iran	250	10.8	—	—	CS	MOL	*C. parvum*, *C. hominis*, and *C. meleagridis*
Huibers, 2018	UC	Malawi	35	11	—	—	CS	MOL	—
Ninama, 2018	2013–2016	India	70	48.6	—	—	CS	MIC	—
Umar, 2018	UC	Nigeria	500	37.8	—	—	CS	MIC	—
Uchenna, 2018	UC	Nigeria	49	18.4	—	—	CS	MOL	—
Opoku, 2018	2012–2013	Ghana	50	46	—	—	CS	MIC	—
Gebre, 2019	2016–2017	Ethiopia	384	9.6	—	—	CS	MIC	—
Gebrecherkos, 2019	2015–2016	Ethiopia	150	6.7	—	—	CS	MIC	—
Gebrewahid, 2019	2017	Ethiopia	242	6.3	—	—	CS	MIC	—
Udeh, 2019	2018	Nigeria	891	2.1	150	0	CC	MIC	*C. parvum*
Sannella, 2019	1999–2004	Thailand	155	59.4	—	—	CS	MOL	*C. hominis* (laA16R3, laA18R3, laA19R3, laA20R3, lbA9G3, ldA11, ldA17, leA11G3T3, lfA12G1), *C. meleagridis* (lllb, llleA22G1R1, lllgA19G3R1), *C. canis*, *C. felis*, *C. suis*, and *C. parvum* (lloA16G1)
Nakibirango, 2019	2018	Uganda	138	2.2	—	—	CS	MIC	—
Namakula, 2019	2019	Uganda	150	18.7	—	—	CS	MIC	—
Mbiandou, 2019	2013–2015	Cameroon	283	7.1	245	1.2	CC	MIC	—
Abange, 2020	2018	Cameroon	119	5.9	95	0	CC	MIC	—
Belay, 2020	2018	Ethiopia	112	8	242	0	CC	MIC	*C. parvum*
Dawet, 2020	2016–2017	Nigeria	100	4	—	—	CS	MIC	—
Irawati, 2020	UC	Indonesia	50	4	—	—	CS	MOL	—
Yulfi, 2020	2018–2019	Indonesia	54	24.1	—	—	CS	MIC	—
Sinyangwe, 2020	2015–2016	Zambia	326	9.5	—	—	CS	MIC	—
Najafi‐Asl, 2020	2018–2019	Iran	133	0	—	—	CS	MIC	—
Lengongo, 2020	2016	Gabon	115	0.9	68	0	CC	MIC	—
Yitbarek, 2021	2019–2020	Ethiopia	384	8.6	—	—	CS	MIC	—
Umoru, 2021	UC	Nigeria	400	19.8	—	—	CS	SER^g^	—
Irawati, 2021	UC	Indonesia	40	5	—	—	CS	MIC	—
Getachew, 2021	2018	Ethiopia	304	19.7	—	—	CS	MIC	—
Betancourth, 2021	2019–2020	Honduras	102	3.9	—	—	CS	MOL	*C. parvum* (llaA15G2R1)
Botero‐Garces, 2021	2018–2019	Colombia	192	0.5	—	—	CS	MIC	—
Chandi, 2021	2017–2020	India	80	6.3	—	—	CS	MIC	*C. parvum*
Dahal, 2021	2019	Nigeria	100	13	—	—	CS	MOL	*C. parvum*
Dankwa, 2021	2018–2019	Ghana	418	6.2	—	—	CS	SER	—
Fumilayo, 2021	UC	Nigeria	96	54.2	—	—	CS	MIC	—
Diptyanusa, 2022	2021	Indonesia	52	42.3	—	—	CS	MOL	—
Zhao, 2022	2016–2017	China	384	3.4	199	0	CC	MOL	*C. hominis* (IbA20G2, Ia18R4), *C. meleagridis* (IIIbA23G1R1, IIIeA15G2R1, IIIgA26G1R1), and *C. cuniculus*
Tanko, 2022	UC	Nigeria	183	4.9	183	0.5	CC	SER	—
Tankoua‐Tchounda, 2022	2021	Cameroon	240	4.6	—	—	CS	MIC	—
Feleke, 2022	2019	Ethiopia	223	1.8	—	—	CS	MIC	—
Fiacre‐Tanguy, 2022	2018–2020	Ivory Coast	363	5	—	—	CS	MOL	—
Ifeoma, 2022	2019–2020	South Africa	600	1.6	—	—	CS	MIC	—
Makwana, 2022	UC	India	100	12	—	—	CS	MIC	*C. parvum*
Mesfun, 2022	UC	Ethiopia	163	7.4	—	—	CS	MIC	—
Mohamed, 2022	2019	Egypt	100	16	—	—	CS	MOL	*C. hominis*, *C. parvum*, *C. meleagridis*, and *C. hominis*/*C. meleagridis*
Muhammad, 2022	UC	Nigeria	110	10	—	—	CS	MIC	—
Sanchez‐Giler, 2022	2013–2014	Ecuador	60	5	—	—	CS	MOL	—
Bejide, 2023	2019–2021	Nigeria	10	40	55	7.3	CC	MOL	*C. parvum*
Gupta, 2023	2023	India	60	48.3	—	—	CS	MIC	—
Harminarti, 2023	2018–2019	Indonesia	40	5	—	—	CS	MIC	—
Ibrahim, 2023	2021–2022	Egypt	70	28.5	—	—	CS	MOL	*C. parvum*
Jiang, 2023	2013–2017	China	155	3.9	—	—	CS	MOL	*C. hominis* (IaA28R4, IeA12G3T3), *C. parvum*, *C. meleagridis*, and *C. andersoni*
Manurung, 2023	2018–2022	Indonesia	253	11.5	—	—	CS	MIC	—
Muhammad, 2023	UC	Nigeria	100	19	—	—	CS	SER	—
Oyakhire, 2023	UC	Nigeria	196	5.1	—	—	CS	MIC	—
Seema, 2023	2020–2023	India	1477	2.8	—	—	CS	MIC	—
Semmani, 2023	2016–2018	Algeria	350	9.4	—	—	CS	MOL	*C. parvum* (llaA14G2R1, llaA15G2R1, llaA16G2R1, llaA20G1R1, llaA21G1R1, lldA16G1, lldA19G1), *C. hominis* (laA24, laA22R2, lbA10G2, lbA13G3), and *C. felis*
Ukibe, 2023	UC	Nigeria	250	7.6	—	—	CS	MIC	—
Waghmare, 2023	2022–2023	India	140	17.9	—	—	CS	MIC	—
Zaldívar‐Lopez, 2023	2020	Mexico	230	72.6	—	—	CS	MIC	—
AbdAllh, 2024	2017	Libya	65	7.7	—	—	CS	MIC	—
Almaw, 2024	2023	Ethiopia	140	11.4	—	—	CS	MIC	—
Ishar, 2024	2015–2016	Nigeria	200	44	100	34	CC	MIC	—
Obebe, 2024	2018–2019	Nigeria	330	7.6	—	—	CS	MIC	—
Sarfo, 2024	UC	Ghana	640	11.4	83	1.2	CC	MOL	—
Zhuhua, 2024	2022–2023	China	382	0.5	—	—	CS	MOL	*C. meleagridis* (IIIeA17G2R1, IIIbA25G1R1a)
Lamine, 2025	2011–2021	Niger Republic	224	30.1	—	—	CS	MIC	—
Mulie, 2025	2019–2020	Ethiopia	384	7.8	—	—	CS	MIC	—
Rashidifar, 2025	UC	Iran	100	9	—	—	CS	MOL	*C. hominis* and *C. parvum*

^a^UC: unclear.

^b^The dashed line (—) indicates that these studies did not have a control group.

^c^CS: cross‐sectional design.

^d^CC: case–control design.

^e^MIC: microscopic detection.

^f^MOL: molecular detection.

^g^SER: serological detection.

### 3.3. Quality Assessment

Of 72 cross‐sectional studies appraised, all were of moderate quality, scoring between 6 and 6.5 due to limitations in addressing confounding factors (checklist items 5 and 6). All 17 case–control studies were of high quality, with scores ranging from 7.5 to 8, demonstrating appropriate case/control definitions, valid outcome assessment, and suitable statistical analyses. The primary weaknesses in the case–control studies were incomplete reporting of matching and insufficient discussion of potential confounding factors (Supporting Table 1).

### 3.4. Pooled Prevalence of *Cryptosporidium* spp. in HIV/AIDS Patients and OR Analysis

**FIGURE 2:**
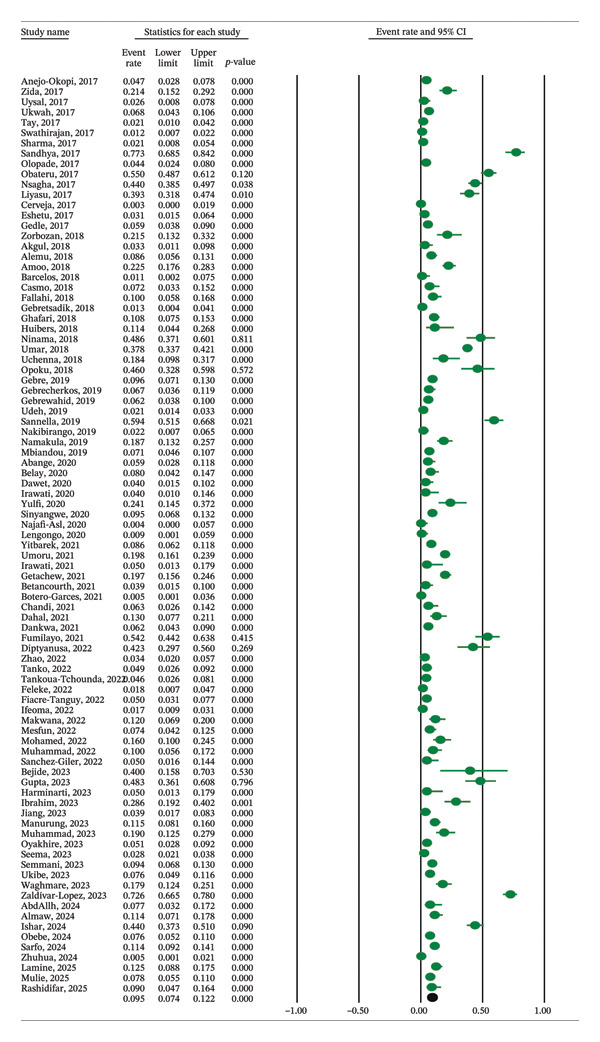
Forest plot showing the prevalence of *Cryptosporidium* spp. in HIV/AIDS patients. Each horizontal green line represents the 95% confidence interval (CI) for the prevalence reported in an individual study. The green circles indicate the point estimate (event rate) for each study. The black circle at the bottom summarizes the pooled prevalence and its 95% CI.

**FIGURE 3:**
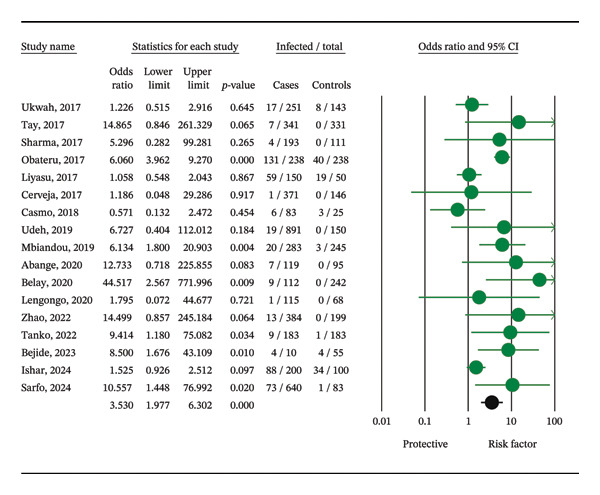
Forest plot showing the weighted random‐effects odds ratio (OR) with 95% confidence intervals (CIs) for *Cryptosporidium* spp. in HIV/AIDS patients compared with healthy controls. Each green circle represents the OR for an individual study. Horizontal lines indicate the 95% CI for each study; wider intervals reflect lower precision due to smaller sample sizes or few positive cases. The vertical solid line at OR = 1 denotes the null value (no association). The black circle at the bottom represents the pooled OR and its 95% CI. OR values to the right of the vertical line indicate increased odds of infection in HIV/AIDS patients (risk factor), while values to the left indicate a potential protective effect.

The overall pooled prevalence of *Cryptosporidium* spp. among HIV‐positive patients was 9.5% (95% CI: 7.4%–12.2%), with substantial heterogeneity (*I* ^2^ = 96.6%) (Figure 2). From 17 case–control studies, HIV‐positive patients had significantly higher odds of *Cryptosporidium* infection compared with HIV‐negative controls (OR = 3.57, 95% CI: 1.97–6.30, *p* < 0.001) (Figure 3).

### 3.5. Subgroup Analyses

Subgroup analyses revealed variation in the prevalence of *Cryptosporidium* spp. among HIV/AIDS patients across multiple study‐level characteristics (Table 2). When stratified by publication year (Supporting Figure 1), pooled prevalence was similar in studies from 2017 to 2020 (9.1%, 95% CI: 6.2–13.2) and 2021–2025 (9.9%, 95% CI: 7.1–13.7). Continental analysis demonstrated that pooled prevalence was highest in Central America (25.3%, 95% CI: 0.6–95.3), followed by Africa (9.8%, 95% CI: 7.5–12.7) and Asia (9.4%, 95% CI: 5.2–16.6), while the lowest estimates were observed in South America (1.7%, 95% CI: 0.4–7.1) (Supporting Figure 2). At the country level (Supporting Figure 3), considerable variability was noted, with very high prevalence in Mexico (72.6%), Thailand (59.4%), Egypt (21.7%), and Burkina Faso (21.4%), contrasted with much lower values in Brazil (1.1%), Colombia (0.5%), Gabon (0.9%), and South Africa (1.7%). Studies with smaller sample sizes (≤ 100 participants) reported higher prevalence (15.3%, 95% CI: 10.5–21.6) compared to larger studies (> 100 participants, 7.9%, 95% CI: 5.8–10.7) (Supporting Figure 4).

**TABLE 2:** Subgroup analysis of *Cryptosporidium* spp. prevalence in people with HIV/AIDS according to publication year, continent, country, country income, study design, diagnostic method, HDI value, and sample size.

Subgroup variable	Prevalence % (95% CI)	Heterogeneity (Q)	No. studies	df (Q)	*I* ^2^ (%)	*p*‐value
*Publication year*						
2017–2020	9.1 (6.2–13.2)	1397.1	45	44	96.8	*p* < 0.05
2021–2025	9.9 (7.1–13.7)	1114.4	44	43	96.1	*p* < 0.05

*Continent*						
Africa	9.8 (7.5–12.7)	1466.1	58	57	96.1	*p* < 0.05
Asia	9.4 (5.2–16.6)	797.9	26	25	96.9	*p* < 0.05
Central America	25.3 (0.6–95.3)	61.7	2	1	98.4	*p* < 0.05
South America	1.7 (0.4–7.1)	4.6	3	2	56.8	*p* > 0.05

*Sample size*						
≤ 100	15.3 (10.5–21.6)	237.6	26	25	89.5	*p* < 0.05
> 100	7.9 (5.8–10.7)	2327.1	63	62	97.3	*p* < 0.05

*Country*						
Algeria	9.4 (6.8–13)	0	1	0	0	*p* > 0.05
Brazil	1.1 (0.2–7.5)	0	1	0	0	*p* > 0.05
Burkina Faso	21.4 (15.2–29.2)	0	1	0	0	*p* > 0.05
Cameroon	10.5 (2.2–37.7)	152.6	4	3	98.1	*p* < 0.05
China	2.3 (0.9–5.6)	6.8	3	2	70.7	*p* > 0.05
Colombia	0.5 (0.1–3.6)	0	1	0	0	*p* > 0.05
Ecuador	5 (1.6–14.4)	0	1	0	0	*p* > 0.05
Egypt	21.7 (11.8–36.4)	3.8	2	1	73.8	*p* > 0.05
Ethiopia	7 (5.2–9.5)	80.8	14	13	83.9	*p* < 0.05
Gabon	0.9 (0.1–5.9)	0	1	0	0	*p* > 0.05
Ghana	10.2 (3.6–25.4)	77.4	4	3	96.1	*p* < 0.05
Honduras	3.9 (1.5–10)	0	1	0	0	*p* > 0.05
India	16.4 (4.6–44.4)	467.6	8	7	98.5	*p* < 0.05
Indonesia	12.6 (5.7–25.8)	39.8	6	5	87.4	*p* < 0.05
Iran	9.2 (5.8–14.2)	6	4	3	50	*p* > 0.05
Ivory Coast	5 (3.1–7.7)	0	1	0	0	*p* > 0.05
Libya	7.7 (3.2–17.2)	0	1	0	0	*p* > 0.05
Malawi	11.4 (4.4–26.8)	0	1	0	0	*p* > 0.05
Mexico	72.6 (66.5–78)	0	1	0	0	*p* > 0.05
Mozambique	1.6 (0.1–30.5)	9.5	2	1	89.5	*p* < 0.05
Nepal	2.1 (0.8–5.4)	0	1	0	0	*p* > 0.05
Niger Republic	12.5 (8.8–17.5)	0	1	0	0	*p* > 0.05
Nigeria	14.5 (9.3–22)	679.7	21	20	97	*p* < 0.05
South Africa	1.7 (0.9–3.1)	0	1	0	0	*p* > 0.05
Thailand	59.4 (51.5–66.8)	0	1	0	0	*p* > 0.05
Turkey	6.3 (1.3–26.2)	18.5	3	2	89.2	*p* < 0.05
Uganda	7.1 (0.8–42.8)	14.2	2	1	92.9	*p* < 0.05
Zambia	9.5 (6.8–13.2)	0	1	0	0	*p* > 0.05

*HDI value* ^a^						
High	7.5 (3.9–13.9)	691.7	24	23	96.7	*p* < 0.05
Low	7.5 (5.6–10)	117.1	19	18	84.6	*p* < 0.05
Medium	12.3 (8.6–17.2)	1604.2	43	42	97.4	*p* < 0.05
Very high	6.3 (1.3–26.2)	18.5	3	2	89.2	*p* < 0.05

*Study design*						
CC^b^	7.5 (3.7–14.5)	629.3	17	16	97.5	*p* < 0.05
CS^c^	10.1 (7.6–13.1)	1971.6	72	71	96.4	*p* < 0.05

*Country income*						
Low	7.6 (5.7–10)	136.6	21	20	85.4	*p* < 0.05
Lower‐middle	12.9 (9.1–18)	1591.4	43	42	97.4	*p* < 0.05
Upper‐middle	6.6 (3.4–12.4)	714.4	25	24	96.6	*p* < 0.05

*Diagnostic method*						
MIC^d^	9.4 (6.8–12.9)	2134.9	61	60	97.2	*p* < 0.05
MOL^e^	9.5 (6.1–14.4)	371.8	24	23	93.8	*p* < 0.05
SER^f^	10.8 (5.2–21.2)	43.8	4	3	93.1	*p* < 0.05

^a^Human Development Index (HDI) values above 0.800 are classified as very high, those between 0.700 and 0.799 as high, from 0.550 to 0.699 as medium, and below 0.550 as low.

^b^CC: case–control studies.

^c^CS: cross‐sectional studies.

^d^MIC: microscopic detection.

^e^MOL: molecular detection.

^f^SER: serological detection.

Stratification by HDI showed that prevalence was highest in medium HDI countries (12.3%, 95% CI: 8.6–17.2), moderate in low HDI countries (7.5%, 95% CI: 5.6–10.0), and lowest in high or very high HDI settings (7.5% and 6.3%, respectively) (Supporting Figure 5). By study design (Supporting Figure 6), cross‐sectional studies reported a higher prevalence (10.1%, 95% CI: 7.6–13.1) compared to case–control studies (7.5%, 95% CI: 3.7–14.5). Regarding country income level (Supporting Figure 7), the highest prevalence was detected in lower‐middle‐income countries (12.9%, 95% CI: 9.1–18.0), followed by low‐income countries (7.6%, 95% CI: 5.7–10.0), while upper‐middle‐income countries had the lowest prevalence (6.6%, 95% CI: 3.4–12.4). The diagnostic method did not strongly influence prevalence estimates, with microscopy (9.4%), molecular assays (9.5%), and serology (10.8%) yielding comparable results (Supporting Figure 8).

### 3.6. Genetic Diversity of *Cryptosporidium* spp. in HIV/AIDS Patients

Among the 24 molecular studies included in this systematic review, only 14 reported species distribution and subtypes of *Cryptosporidium* spp. in HIV/AIDS patients. Overall, the species identified included *C. hominis*, *C. parvum*, *C. meleagridis*, *C. felis*, *C. viatorum*, *C. canis*, *C. suis*, *C. andersoni*, and *C. cuniculus* (Table 1).

*C. hominis* was the most frequently reported species, with several subtypes identified, including laA22R2, IaA16R3, IaA18R3, IaA19R3, IaA20R3, IaA23R3, laA24, IaA29R3, IaA27R4, IaA28R4, Ia18R4, IbA9G3, IbA10G2, IbA20G2, lbA13G3, IdA11, IdA17, IeA11G3T3, IeA12G3T3, and IfA12G1. *C. parvum* was the second most prevalent species, with subtypes IIcA5G3b, IIcA5G3d, IIcA5G3k, IIeA10G1, IIoA16G1, IIaA14G2R1, IIaA15G2R1, IIaA16G2R1, IIaA20G1R1, IIaA21G1R1, IIdA16G1, and IIdA19G1. *C. meleagridis* subtypes included IIIbA23G1R1, IIIbA25G1R1a, IIIeA15G2R1, IIIeA17G2R1, IIIeA22G1R1, IIIgA19G3R1, and IIIgA26G1R1. Less frequently reported species were *C. felis*, *C. viatorum*, *C. canis*, *C. andersoni*, *C. suis*, and *C. cuniculus*, each identified in one or more studies. Only one study reported co‐infections, such as *C. hominis*/*C. meleagridis* (Table 1).

### 3.7. Pool of Positive *Cryptosporidium* spp. in HIV/AIDS Patients by Age, Sex, CD4 Count, and Diarrhea

Data from Table 3 highlighted the distribution of positive cases according to demographic and clinical variables, though these were calculated only within the pool of positives rather than as true subgroup prevalence. Among HIV/AIDS patients positive for *Cryptosporidium* spp., 39.8% were aged ≤ 30 years and 64.7% were aged > 30 years. Female patients accounted for a slightly higher proportion of positives (54.5%) compared to males (45.8%). The majority of positive cases had CD4 counts ≤ 200 cells/μL (62.5%), while only 43.2% had counts above 200. Finally, 78.4% of positives presented with diarrhea, compared to 43.8% of those without diarrhea (Supporting Figures 9–12).

**TABLE 3:** Distribution of positive *Cryptosporidium* spp. cases in HIV/AIDS patients, categorized by age, sex, CD4 T cell count, and diarrhea.

Variables	Prevalence % (95% CI)^a^	No. datasets^b^	df (Q)	Heterogeneity (Q)	*I* ^2^
*Age groups (Y)*					
≤ 30	39.8 (30.9–49.4)	19	18	62.1	71
> 30	64.7 (56.6–72)	22	21	52.4	59.9

*Sex*					
Female	54.5 (48.5–60.5)	31	30	60.8	50.7
Male	45.8 (39.8–51.9)	32	31	62.8	50.6

*CD4 T cell count*					
≤ 200	62.5 (53.7–70.6)	35	34	133.8	74.6
> 200	43.2 (33.8–53.1)	31	30	145.1	79.3

*Symptoms*					
Diarrhea	78.4 (67.1–86.5)	30	29	152.2	80.9
Nondiarrhea	43.8 (30.3–58.3)	19	18	99	81.8

^a^Values represent the proportion of positives within each category (age, sex, CD4 count, and diarrhea) relative to the total number of positives, not true subgroup prevalence, since denominators (total tested per category) were not consistently reported.

^b^The variation in the number of datasets is due to the fact that the evaluated HIV/AIDS patients in some studies did not include all classifications in one variable. For example, in certain studies, either all HIV/AIDS patients or only positive cases belonged to a specific age group (only < 30 or > 30). This makes it difficult to accurately assess the proportion of positive cases by examined variables, and therefore the results should be interpreted with caution.

### 3.8. Sensitivity Analysis

Sequential exclusion of individual studies did not materially alter the overall prevalence estimate, indicating the robustness of the findings (Supporting Figure 13).

### 3.9. Meta‐Regression

Meta‐regression analyses were performed to explore potential sources of heterogeneity using four continuous covariates: sample size, year of publication, HDI, and country income level (Figure 4). Among these variables, sample size showed a statistically significant negative association with the prevalence of *Cryptosporidium* infection in HIV/AIDS patients (coefficient = −0.0019, *p* = 0.003), indicating that studies with larger sample sizes tended to report lower prevalence estimates. The inclusion of this covariate reduced the between‐study variance from *τ* ^2^ = 1.6218 to *τ* ^2^ = 1.4388 and the *I* ^2^ value from 96.62% to 96.15%, with an *R* ^2^ analog of 0.11, suggesting that approximately 11% of the observed heterogeneity was explained by sample size. By contrast, year of publication (coefficient = 0.0073, *p* = 0.897), HDI (coefficient = −0.3848, *p* = 0.764), and country income (coefficient ≈ 0.0000, *p* = 0.230) were not statistically significant predictors of prevalence. The inclusion of these covariates did not meaningfully reduce *τ* ^2^ or *I* ^2^, and their *R* ^2^ analog values (0.00–0.03) indicated negligible explanatory power.

FIGURE 4Meta‐regression bubble plots show the effect of sample size (a), publication year (b), HDI value (c), and country income (d) on *Cryptosporidium* spp. prevalence in HIV/AIDS patients. Bubble size reflects study weight in the meta‐analysis. The regression line indicates the relationship between the covariate and prevalence. Sample size had a significant positive association (*p* = 0.01), while publication year, HDI value, and country income showed no significant effects, suggesting they did not explain between‐study heterogeneity.

**Figure:**
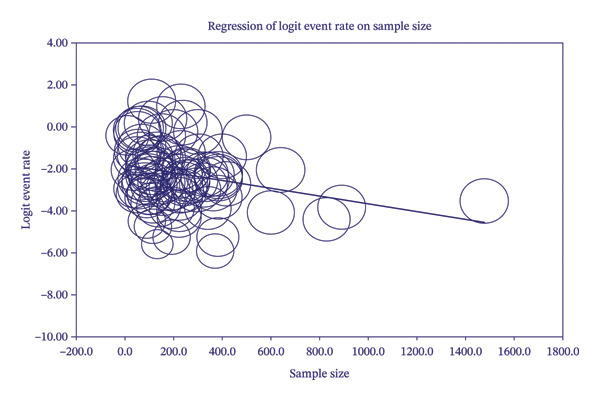
(a)

**Figure:**
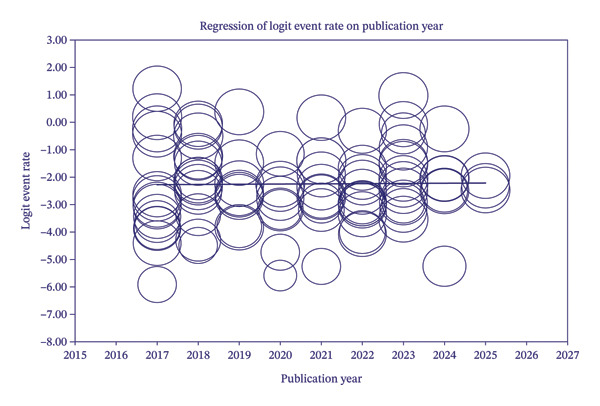
(b)

**Figure:**
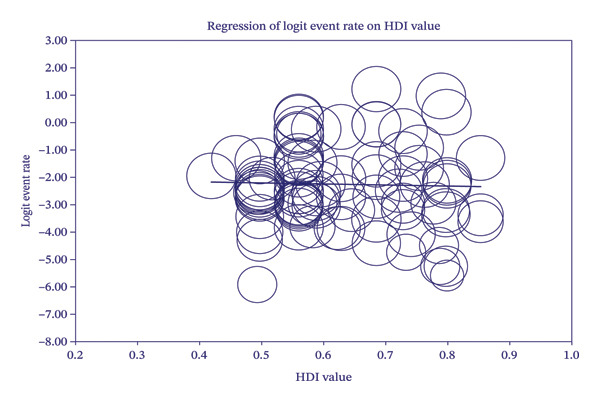
(c)

**Figure:**
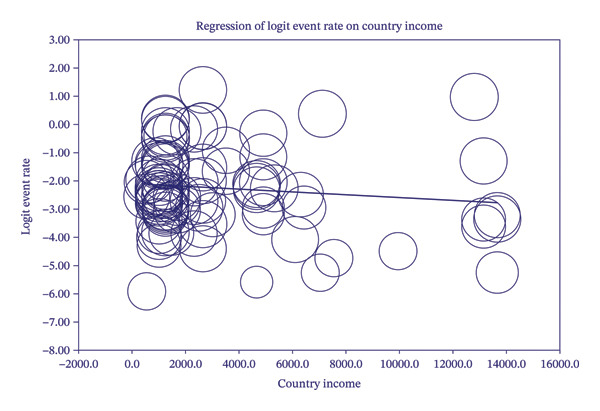
(d)

### 3.10. Publication Bias

**FIGURE 5:**
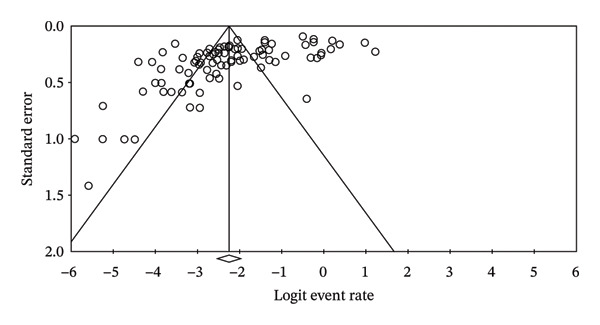
Funnel plot illustrating publication bias in the current systematic review and meta‐analysis. The *x*‐axis represents the logit event rate, while the *y*‐axis displays the standard error. The plot’s symmetry is an indicator of potential publication bias, with smaller studies being dispersed at the bottom and larger studies toward the top. The triangles represent the expected distribution of studies under the assumption of no bias. Any asymmetry observed may suggest selective reporting or missing studies, potentially affecting the validity of the findings.

Visual inspection of funnel plots suggested potential asymmetry, and Egger’s test confirmed evidence of publication bias (*p* < 0.001) (Figure 5).

## 4. Discussion

In the present meta‐analysis, the pooled prevalence of *Cryptosporidium* spp. among HIV/AIDS patients, based on 89 included studies, was 9.5% (95% CI: 7.4%–12.2%). Furthermore, the analysis of 17 case–control studies indicated that HIV‐positive individuals were 3.5 times more likely to be infected with *Cryptosporidium* spp. compared to HIV‐negative controls, a statistically significant association (OR = 3.53, 95% CI: 1.97–6.30, *p* < 0.001). For comparison, a prior systematic review and meta‐analysis covering studies up to 2017 reported an overall prevalence of 11.2% (95% CI: 9.4%–13%) among HIV‐positive patients [16], suggesting a modest decline in reported prevalence in more recent studies, which may reflect improvements in ART coverage, public health interventions, or differences in diagnostic approaches. In contrast, a meta‐analysis of *Cryptosporidium* spp. in the general healthy population reported a pooled prevalence of 7.6% [10], indicating that HIV‐positive patients remain at substantially higher risk. Although HIV/AIDS patients are generally more vulnerable to infections, the risk of *Cryptosporidium* spp. is influenced by factors such as the degree of immunosuppression, exposure to contaminated sources, poor hygiene and nutrition, and limited access to healthcare.

Evidence from other immunocompromised populations also supports the notion of increased vulnerability. For example, adult kidney transplant recipients and cancer patients exhibit higher prevalence and odds of *Cryptosporidium* spp. compared with healthy controls, highlighting the role of systemic immunosuppression across diverse patient groups [109]. Similarly, analyses of other intestinal parasites in HIV/AIDS patients demonstrate comparable patterns: the pooled prevalence of *Giardia duodenalis* was 5%, with HIV‐positive patients showing a 1.7‐fold higher odds of infection than controls [110], while *Cyclospora cayetanensis* weighted prevalence reached 4%, yielding an OR of 3.5 [111]. Among immunocompromised non‐HIV populations, the prevalence and odds were often even higher, as seen with ORs of 5.4 for *C. cayetanensis* infection [112]. These comparisons emphasize that immunodeficiency, whether due to HIV, immunosuppressive therapy, or organ transplantation, consistently increases susceptibility to enteric protozoan infections. The findings from *Blastocystis* sp. studies further illustrate this trend [113]. The pooled prevalence among immunocompromised patients was 10.3%, with the highest prevalence observed in hemodialysis (16.1%) and cancer patients (12.5%), whereas HIV/AIDS patients had a prevalence of 8.4%. Case–control analyses revealed significantly elevated odds in these groups, particularly cancer and hemodialysis patients, reinforcing the general principle that immunosuppression amplifies the risk of intestinal parasitic infections. Taken together, the current results align with these patterns: HIV‐positive patients demonstrate both higher prevalence and significantly increased odds of *Cryptosporidium* spp. compared with immunocompetent individuals. However, the slightly lower prevalence compared with earlier reviews may reflect heterogeneity in study designs, number of studies included, geographic distribution, sample sizes, diagnostic methods, and local public health conditions. Notably, the consistent risk elevation across diverse immunocompromised populations underscores the need for targeted preventive strategies, enhanced diagnostic surveillance, and improved water and sanitation interventions to reduce the burden of cryptosporidiosis and other intestinal parasitic infections in vulnerable populations.

In the present study, the *I* ^2^ value of 96.6% indicates near‐total heterogeneity among the included studies. This means that the observed variation in prevalence estimates is not due to random sampling but to fundamental differences between studies (e.g., populations, diagnostic methods, and intensity of local transmission). Such high heterogeneity calls into question the very relevance of calculating a single “mean” prevalence. The pooled estimate of 9.5% masks extreme variability and should be considered not as a globally representative value but as an arithmetic mean of highly disparate results.

In the current study, the molecular data highlight considerable genetic diversity of *Cryptosporidium* spp. among HIV/AIDS patients. Consistent with global epidemiological patterns [13] and with systematic reviews and meta‐analyses conducted on HIV/AIDS patients in certain African regions, such as Nigeria [114], *C. hominis* and *C. parvum* were the predominant species, reflecting their primary roles in human infection, with *C. hominis* typically anthroponotic and *C. parvum* zoonotic in nature. The extensive variety of subtypes within these species indicates multiple circulating strains and potential for diverse transmission routes. In the present study, the detection of other species, including *C. meleagridis*, *C. felis*, *C. viatorum*, *C. canis*, *C. andersoni*, *C. suis*, and *C. cuniculus*, underscores the zoonotic potential and broad host range of *Cryptosporidium* spp. Such diversity suggests frequent cross‐species transmission and environmental exposure, particularly in regions with close human–animal interactions or inadequate water and sanitation infrastructure. However, these molecular findings should be interpreted with caution. Some studies reporting *C. parvum* relied on microscopy‐based identification, which has inherent limitations and may misclassify species due to morphological similarities among oocysts. Only a minority of studies performed genotyping or subtyping, limiting the precision of species distribution data. Furthermore, small sample sizes and regional variability may have biased the observed diversity. Despite these limitations, the overall pattern indicates that while *C. hominis* and *C. parvum* are dominant in HIV/AIDS patients, other zoonotic species and diverse subtypes are consistently detected, emphasizing the complex epidemiology of cryptosporidiosis in immunocompromised populations. Future research should prioritize molecular diagnostics, larger sample sizes, and systematic subtyping to better elucidate transmission dynamics, assess zoonotic risk, and guide targeted prevention strategies.

The subgroup analyses provide important insights into the epidemiology of cryptosporidiosis in HIV/AIDS patients but also reveal substantial heterogeneity that must be interpreted with caution. The similarity of prevalence between the two publication periods (2017–2020 vs. 2021–2025) suggests that despite improvements in ART coverage and water sanitation programs in many regions, the burden of *Cryptosporidium* spp. in HIV/AIDS patients has remained stable over the past 8 years. This stability indicates the persistence of structural risk factors, such as inadequate water treatment, environmental contamination, and zoonotic exposure, that are not easily mitigated by general healthcare improvements.

The highest pooled prevalence of *Cryptosporidium* spp. among HIV/AIDS patients was observed in Central America (25.3%, based on two studies) and Africa (9.8%, based on 58 studies), although the estimate for Asia (9.4%, based on 26 studies) was comparable to that of Africa. The markedly elevated pooled prevalence reported for Central America is likely influenced by the limited geographical representation and the disproportionately high prevalence reported in individual studies, such as the 72.6% estimate from Mexico, which can substantially inflate aggregated values in meta‐analytic models. In contrast, the elevated pooled prevalence observed in Africa reflects data from 58 studies conducted across 15 African countries, suggesting that this pattern is not attributable to limited datasets but rather to structural determinants, including inadequate water and sanitation infrastructure, restricted access to healthcare services, widespread poverty, and increased environmental exposure risks. More broadly, although the overall number of studies available from some continents, particularly Africa and Asia, is relatively large, country‐level data remain sparse, with many countries represented by only a single study in the present review. This scarcity at the national level limits the ability to generalize findings to individual countries and underscores the need for cautious interpretation of country‐specific prevalence estimates.

The strong effect of sample size observed in the subgroup analysis is consistent with the meta‐regression findings: smaller studies reported substantially higher prevalence compared to larger studies. This may reflect small‐study effects, publication bias, or a tendency for smaller investigations to be conducted in high‐risk populations or using more sensitive diagnostic tools. The association of higher prevalence with medium HDI and lower‐middle‐income countries supports the hypothesis that limited resources and infrastructure exacerbate susceptibility to waterborne infections, although again the wide heterogeneity indicates that socioeconomic indicators alone cannot fully explain the burden. Interestingly, the diagnostic method did not significantly influence prevalence estimates, with microscopy, molecular, and serological methods producing similar results. This may be partly due to the limited number of serology‐based studies and the variability of molecular methods used. In addition, microscopy‐based results may underestimate prevalence due to lower sensitivity, while molecular studies may capture more subclinical infections, yet these differences did not manifest strongly in pooled estimates.

The demographic and clinical distributions from Table 3 offer further insight into the profile of HIV/AIDS patients with *Cryptosporidium* spp. A larger proportion of positives were above 30 years of age, suggesting that adult patients remain highly susceptible, possibly due to cumulative exposure and immunological decline. The slightly higher proportion of females compared to males could reflect social and behavioral factors, though this difference was modest. Importantly, the clear association with immune suppression was evident: Nearly two‐thirds of positives had CD4 counts ≤ 200 cells/μL, consistent with the role of cellular immunity in controlling cryptosporidiosis. Likewise, the majority of positive patients presented with diarrhea, reinforcing its role as a hallmark clinical manifestation in HIV‐associated cryptosporidiosis. Nevertheless, these analyses are constrained by significant limitations. The demographic and clinical variables in Table 3 were calculated as proportions of positive cases only, not as true prevalence within the entire study population, because denominators were inconsistently reported. As a result, these findings provide useful descriptive information but cannot be directly compared across studies or generalized to all HIV/AIDS patients. Similarly, several subgroup categories, particularly those at the country and continental level, were based on few or single studies, making those estimates highly unstable.

The findings of the present meta‐analysis provide important insights into the epidemiology of *Cryptosporidium* spp. among HIV/AIDS patients and highlight several methodological and epidemiological considerations. The meta‐regression analysis revealed that sample size was the only significant covariate associated with the reported prevalence of infection. Specifically, smaller studies tended to report substantially higher prevalence rates, whereas larger studies yielded lower estimates. This inverse association was statistically significant, and sample size explained approximately 11% of the observed heterogeneity. The subgroup analyses corroborated this finding, showing that studies with ≤ 100 participants reported nearly threefold higher prevalence compared to those with larger sample sizes. Taken together, these results strongly suggest the presence of a small‐study effect, whereby smaller investigations, often conducted in high‐burden regions, or employing more sensitive diagnostic tools tend to overestimate prevalence relative to larger, more representative studies.

Sensitivity analysis further supported the robustness of the pooled prevalence estimate, as the sequential exclusion of individual studies did not significantly alter the overall findings. Nevertheless, the high level of heterogeneity persisted, indicating that additional unmeasured variables, such as patient immune status (e.g., CD4+ T‐cell counts, ART coverage), local environmental conditions, diagnostic approaches, and geographical differences in species distribution, may contribute to the wide variation in reported prevalence. The presence of small‐study effects was also confirmed by publication bias assessments. The funnel plot demonstrated asymmetry, and Egger’s regression test indicated statistically significant bias, suggesting that smaller studies with higher prevalence rates were more likely to be published. This observation is consistent with the meta‐regression and subgroup findings and reinforces the interpretation that methodological differences rather than true epidemiological variation may partly drive the observed prevalence patterns.

Interestingly, other covariates examined in the meta‐regression, year of publication, HDI, and country income level did not show statistically significant associations with prevalence. This lack of association implies that the burden of cryptosporidiosis among HIV/AIDS patients remains relatively constant across different socioeconomic contexts and has not declined meaningfully over the study period despite improvements in ART coverage and water and sanitation infrastructures in many regions. It is also possible that national‐level indicators such as HDI and income fail to capture within‐country disparities in water access, healthcare availability, and exposure to zoonotic sources, which may obscure true associations at the community level. Overall, the combined evidence from the sensitivity analysis, subgroup analyses, meta‐regression, and publication bias assessments underscores the complexity of interpreting prevalence estimates of cryptosporidiosis in HIV/AIDS patients. While methodological factors such as sample size clearly influence the reported rates, the consistently high heterogeneity highlights the multifactorial nature of infection risk. These findings emphasize the need for larger, standardized, and geographically diverse studies using sensitive diagnostic methods, along with more detailed reporting of patient‐level and setting‐specific characteristics, to generate more accurate and generalizable prevalence estimates in this vulnerable population.

Although this systematic review and meta‐analysis provides comprehensive and up‐to‐date insights into the prevalence, genetic diversity, and risk factors of *Cryptosporidium* spp. in HIV/AIDS patients, several limitations should be acknowledged. First, significant heterogeneity was observed across studies, which was only partially explained by subgroup and meta‐regression analyses. Factors such as variation in study design, sample size, diagnostic methods, and geographical coverage likely contributed to this variability. Second, in some subgroup analyses, particularly those stratified by individual countries or continents, estimates were based on a limited number of studies or even single datasets, thereby restricting the generalizability of these findings. Third, demographic and clinical variables such as age, sex, CD4 T‐cell count, and diarrheal status were inconsistently reported, and in most cases, denominators were missing; hence, analyses for these variables were descriptive rather than true subgroup prevalence comparisons. Fourth, publication bias was detected, indicating that smaller studies with higher prevalence were more likely to be published, which may have inflated pooled estimates. Finally, reliance on microscopy in a proportion of included studies may have led to misclassification or underestimation of species distribution compared with molecular approaches. These limitations highlight the need for cautious interpretation of the results and call for more standardized, large‐scale, and high‐quality epidemiological studies.

Preventive measures against cryptosporidiosis in HIV/AIDS patients should focus on improving access to safe drinking water, strengthening sanitation and hygiene practices, and reducing exposure to potential zoonotic sources. In clinical practice, early initiation and adherence to ART are essential to preserve immune function and reduce susceptibility to opportunistic infections. Routine screening for *Cryptosporidium* spp. in symptomatic HIV/AIDS patients, particularly those with low CD4 counts or persistent diarrhea, should be encouraged to facilitate timely diagnosis and management.

## 5. Conclusion

Despite the statistical limitations of this study, its main message remains valid: Cryptosporidiosis is a significant threat to HIV‐positive patients. Clinicians should maintain a high index of suspicion for this infection, particularly in patients presenting with diarrhea, especially if their CD4 count is 200 cells/μL or lower. This study is a valuable contribution to the literature, confirming that cryptosporidiosis continues to represent a significant burden of disease for people living with HIV/AIDS worldwide. The study successfully synthesizes recent data and highlights the complexity of anthroponotic and zoonotic transmission routes. However, it is imperative to emphasize that its precise quantitative estimates, particularly the pooled prevalence of 9.5%, should be interpreted with extreme caution. The massive heterogeneity between studies and the confirmed presence of significant publication bias mean that this figure is likely an overestimate and does not reflect a uniform epidemiological reality. The main value of this study, therefore, lies not in a single figure but in its qualitative confirmation of the problem’s persistent clinical importance. Moreover, it strikingly highlights methodological gaps and unmet needs in current research. It thus underscores the urgent need for higher‐quality, larger‐scale, and more standardized epidemiological studies to effectively inform public health strategies and the clinical management of cryptosporidiosis.

## Author Contributions

Ali Asghari and Farzad Mahdavi conceived and designed the study. Farzad Mahdavi, Shayan Heidari, Roumina Norouzi, Ali Pouryousef, Mohammad Reza Mohammadi, Kambiz Karimi, Asma Mousivand, and Mahtab Mehboodi had a role in data extraction and methodology. Ali Asghari and Farzad Mahdavi performed the data analysis. Ali Asghari, Farzad Mahdavi, Mohammad Reza Mohammadi, and Ali Pouryousef wrote the manuscript. Ali Asghari, Mohammad Reza Mohammadi, and Hassan Nourmohammadi critically revised the manuscript.

## Funding

This research received no funding.

## Disclosure

All the authors have read and approved the final manuscript.

## Ethics Statement

The authors have nothing to report.

## Conflicts of Interest

The authors declare no conflicts of interest.

## Supporting Information

Additional supporting information can be found online in the Supporting Information section.

## Supporting information


**Supporting Information 1** Supporting Figure 1. The pooled prevalence of *Cryptosporidium* spp. in HIV/AIDS patients based on publication year. Green indicates the prevalence from each study, while orange shows the overall weighted prevalence.

**Supporting Information 2** Supporting Figure 2. The pooled prevalence of *Cryptosporidium* spp. in HIV/AIDS patients based on continent. Green indicates the prevalence from each study, while orange shows the overall weighted prevalence.

**Supporting Information 3** Supporting Figure 3. The pooled prevalence of *Cryptosporidium* spp. in HIV/AIDS patients based on country. Green indicates the prevalence from each study, while orange shows the overall weighted prevalence.

**Supporting Information 4** Supporting Figure 4. The pooled prevalence of *Cryptosporidium* spp. in HIV/AIDS patients based on sample size. Green indicates the prevalence from each study, while orange shows the overall weighted prevalence.

**Supporting Information 5** Supporting Figure 5. The pooled prevalence of *Cryptosporidium* spp. in HIV/AIDS patients based on HDI value. Green indicates the prevalence from each study, while orange shows the overall weighted prevalence.

**Supporting Information 6** Supporting Figure 6. The pooled prevalence of *Cryptosporidium* spp. in HIV/AIDS patients based on study design. Green indicates the prevalence from each study, while orange shows the overall weighted prevalence.

**Supporting Information 7** Supporting Figure 7. The pooled prevalence of *Cryptosporidium* spp. in HIV/AIDS patients based on country income level. Green indicates the prevalence from each study, while orange shows the overall weighted prevalence.

**Supporting Information 8** Supporting Figure 8. The pooled prevalence of *Cryptosporidium* spp. in HIV/AIDS patients based on the diagnostic method. Green indicates the prevalence from each study, while orange shows the overall weighted prevalence.

**Supporting Information 9** Supporting Figure 9. Pool of positive *Cryptosporidium* spp. in HIV/AIDS patients by age. Green indicates the prevalence from each study, while orange shows the overall weighted prevalence.

**Supporting Information 10** Supporting Figure 10. Pool of positive *Cryptosporidium* spp. in HIV/AIDS patients by sex. Green indicates the prevalence from each study, while orange shows the overall weighted prevalence.

**Supporting Information 11** Supporting Figure 11. Pool of positive *Cryptosporidium* spp. in HIV/AIDS patients by CD4 T‐cell count. Green indicates the prevalence from each study, while orange shows the overall weighted prevalence.

**Supporting Information 12** Supporting Figure 12. Pool of positive *Cryptosporidium* spp. in HIV/AIDS patients with diarrhea. Green indicates the prevalence from each study, while orange shows the overall weighted prevalence.

**Supporting Information 13** Supporting Figure 13. Sensitivity analysis assessing the influence of individual studies on pooled estimates.

**Supporting Information 14** Supporting Table 1. JBI critical appraisal checklist applied for included studies.

## Data Availability

Data are available in the article’s supporting materials.
