# Supplementary material for: Prevalence, Genetic Diversity, and Risk Factors of Cryptosporidium spp. in HIV/AIDS Patients: An Updated Systematic Review and Meta‐Analysis (2017–2025)
Source: Can J Infect Dis Med Microbiol. 2026 Jun 12;2026:2740716. doi: 10.1155/cjid/2740716 (PMC13263408; doi:10.1155/cjid/2740716)
Supplement: Supplementary file 10 — Supporting Information 10 Supporting Figure 10. Pool of positive Cryptosporidium spp. in HIV/AIDS patients by sex. Green indicates the prevalence from each study, while orange shows the overall weighted prevalence. [file CJID-2026-2740716-s009.docx]

**Supplementary Fig. 10.** Pool of positive *Cryptosporidium* spp. in HIV/AIDS patients by sex. Green indicates the prevalence from each study, while orange shows the overall weighted prevalence.
