# Supplementary material for: Prevalence, Genetic Diversity, and Risk Factors of Cryptosporidium spp. in HIV/AIDS Patients: An Updated Systematic Review and Meta‐Analysis (2017–2025)
Source: Can J Infect Dis Med Microbiol. 2026 Jun 12;2026:2740716. doi: 10.1155/cjid/2740716 (PMC13263408; doi:10.1155/cjid/2740716)
Supplement: Supplementary file 11 — Supporting Information 11 Supporting Figure 11. Pool of positive Cryptosporidium spp. in HIV/AIDS patients by CD4 T‐cell count. Green indicates the prevalence from each study, while orange shows the overall weighted prevalence. [file CJID-2026-2740716-s010.docx]

**Supplementary Fig. 11.** Pool of positive *Cryptosporidium* spp. in HIV/AIDS patients by CD4 T-cell count. Green indicates the prevalence from each study, while orange shows the overall weighted prevalence.
