# Supplementary material for: Prevalence, Genetic Diversity, and Risk Factors of Cryptosporidium spp. in HIV/AIDS Patients: An Updated Systematic Review and Meta‐Analysis (2017–2025)
Source: Can J Infect Dis Med Microbiol. 2026 Jun 12;2026:2740716. doi: 10.1155/cjid/2740716 (PMC13263408; doi:10.1155/cjid/2740716)
Supplement: Supplementary file 14 — Supporting Information 14 Supporting Table 1. JBI critical appraisal checklist applied for included studies. [file CJID-2026-2740716-s013.docx]

**Supplementary Table1. JBI critical appraisal checklist applied for included studies.**

| **Cross-sectional** | | | | | | | | | | | |
| --- | --- | --- | --- | --- | --- | --- | --- | --- | --- | --- | --- |
| **Author Name/Year** | **Q1** | **Q2** | **Q3** | **Q4** | **Q5** | **Q6** | **Q7** | **Q8** | **Overall Quality** | | |
| Anejo-Okopi, 2017 | Yes | Yes | Yes | Yes | No | No | Yes | Yes | 6 | | |
| Zida, 2017 | Yes | Yes | Yes | Yes | Unclear | No | Yes | Yes | 6.5 | | |
| Uysal, 2017 | Yes | Yes | Yes | Yes | No | No | Yes | Yes | 6 | | |
| Swathirajan, 2017 | Yes | Yes | Yes | Yes | No | No | Yes | Yes | 6 | | |
| Sandhya, 2017 | Yes | Yes | Yes | Yes | Unclear | No | Yes | Yes | 6.5 | | |
| Olopade, 2017 | Yes | Yes | Yes | Yes | No | No | Yes | Yes | 6 | | |
| Nsagha, 2017 | Yes | Yes | Yes | Yes | Unclear | No | Yes | Yes | 6.5 | | |
| Eshetu, 2017 | Yes | Yes | Yes | Yes | No | No | Yes | Yes | 6 | | |
| Gedle, 2017 | Yes | Yes | Yes | Yes | Unclear | No | Yes | Yes | 6.5 | | |
| Zorbozan, 2018 | Yes | Yes | Yes | Yes | No | No | Yes | Yes | 6 | | |
| Akgul, 2018 | Yes | Yes | Yes | Yes | No | No | Yes | Yes | 6 | | |
| Alemu, 2018 | Yes | Yes | Yes | Yes | Unclear | No | Yes | Yes | 6.5 | | |
| Amoo, 2018 | Yes | Yes | Yes | Yes | No | No | Yes | Yes | 6 | | |
| Barcelos, 2018 | Yes | Yes | Yes | Yes | Unclear | No | Yes | Yes | 6.5 | | |
| Fallahi, 2018 | Yes | Yes | Yes | Yes | No | No | Yes | Yes | 6 | | |
| Gebretsadik, 2018 | Yes | Yes | Yes | Yes | Unclear | No | Yes | Yes | 6.5 | | |
| Ghafari, 2018 | Yes | Yes | Yes | Yes | No | No | Yes | Yes | 6 | | |
| Huibers, 2018 | Yes | Yes | Yes | Yes | Unclear | No | Yes | Yes | 6.5 | | |
| Ninama, 2018 | Yes | Yes | Yes | Yes | No | No | Yes | Yes | 6 | | |
| Umar, 2018 | Yes | Yes | Yes | Yes | Unclear | No | Yes | Yes | 6.5 | | |
| Uchenna, 2018 | Yes | Yes | Yes | Yes | No | No | Yes | Yes | 6 | | |
| Opoku, 2018 | Yes | Yes | Yes | Yes | Unclear | No | Yes | Yes | 6.5 | | |
| Gebre, 2019 | Yes | Yes | Yes | Yes | No | No | Yes | Yes | 6 | | |
| Gebrecherkos, 2019 | Yes | Yes | Yes | Yes | Unclear | No | Yes | Yes | 6.5 | | |
| Gebrewahid, 2019 | Yes | Yes | Yes | Yes | No | No | Yes | Yes | 6 | | |
| Sannella, 2019 | Yes | Yes | Yes | Yes | Unclear | No | Yes | Yes | 6.5 | | |
| Nakibirango, 2019 | Yes | Yes | Yes | Yes | No | No | Yes | Yes | 6 | | |
| Namakula, 2019 | Yes | Yes | Yes | Yes | Unclear | No | Yes | Yes | 6.5 | | |
| Dawet, 2020 | Yes | Yes | Yes | Yes | No | No | Yes | Yes | 6 | | |
| Irawati, 2020 | Yes | Yes | Yes | Yes | Unclear | No | Yes | Yes | 6.5 | | |
| Yulfi, 2020 | Yes | Yes | Yes | Yes | No | No | Yes | Yes | 6 | | |
| Sinyangwe, 2020 | Yes | Yes | Yes | Yes | Unclear | No | Yes | Yes | 6.5 | | |
| Najafi-Asl, 2020 | Yes | Yes | Yes | Yes | No | No | Yes | Yes | 6 | | |
| Yitbarek, 2021 | Yes | Yes | Yes | Yes | No | No | Yes | Yes | 6 | | |
| Umoru, 2021 | Yes | Yes | Yes | Yes | Unclear | No | Yes | Yes | 6.5 | | |
| Irawati, 2021 | Yes | Yes | Yes | Yes | No | No | Yes | Yes | 6 | | |
| Getachew, 2021 | Yes | Yes | Yes | Yes | Unclear | No | Yes | Yes | 6.5 | | |
| Betancourth, 2021 | Yes | Yes | Yes | Yes | No | No | Yes | Yes | 6 | | |
| Botero-Garces, 2021 | Yes | Yes | Yes | Yes | Unclear | No | Yes | Yes | 6.5 | | |
| Chandi, 2021 | Yes | Yes | Yes | Yes | No | No | Yes | Yes | 6 | | |
| Dahal, 2021 | Yes | Yes | Yes | Yes | No | No | Yes | Yes | 6 | | |
| Dankwa, 2021 | Yes | Yes | Yes | Yes | Unclear | No | Yes | Yes | 6.5 | | |
| Fumilayo, 2021 | Yes | Yes | Yes | Yes | No | No | Yes | Yes | 6 | | |
| Diptyanusa, 2022 | Yes | Yes | Yes | Yes | Unclear | No | Yes | Yes | 6.5 | | |
| Tankoua-Tchounda, 2022 | Yes | Yes | Yes | Yes | No | No | Yes | Yes | 6 | | |
| Feleke, 2022 | Yes | Yes | Yes | Yes | Unclear | No | Yes | Yes | 6.5 | | |
| Fiacre-Tanguy, 2022 | Yes | Yes | Yes | Yes | No | No | Yes | Yes | 6 | | |
| Ifeoma, 2022 | Yes | Yes | Yes | Yes | Unclear | No | Yes | Yes | 6.5 | | |
| Makwana, 2022 | Yes | Yes | Yes | Yes | No | No | Yes | Yes | 6 | | |
| Mesfun, 2022 | Yes | Yes | Yes | Yes | No | No | Yes | Yes | 6 | | |
| Mohamed, 2022 | Yes | Yes | Yes | Yes | Unclear | No | Yes | Yes | 6.5 | | |
| Muhammad, 2022 | Yes | Yes | Yes | Yes | No | No | Yes | Yes | 6 | | |
| Sanchez-Giler, 2022 | Yes | Yes | Yes | Yes | Unclear | No | Yes | Yes | 6.5 | | |
| Gupta, 2023 | Yes | Yes | Yes | Yes | No | No | Yes | Yes | 6 | | |
| Harminarti, 2023 | Yes | Yes | Yes | Yes | Unclear | No | Yes | Yes | 6.5 | | |
| Ibrahim, 2023 | Yes | Yes | Yes | Yes | No | No | Yes | Yes | 6 | | |
| Jiang, 2023 | Yes | Yes | Yes | Yes | Unclear | No | Yes | Yes | 6.5 | | |
| Manurung, 2023 | Yes | Yes | Yes | Yes | No | No | Yes | Yes | 6 | | |
| Muhammad, 2023 | Yes | Yes | Yes | Yes | Unclear | No | Yes | Yes | 6.5 | | |
| Oyakhire, 2023 | Yes | Yes | Yes | Yes | No | No | Yes | Yes | 6 | | |
| Seema, 2023 | Yes | Yes | Yes | Yes | No | No | Yes | Yes | 6 | | |
| Semmani, 2023 | Yes | Yes | Yes | Yes | Unclear | No | Yes | Yes | 6.5 | | |
| Ukibe, 2023 | Yes | Yes | Yes | Yes | No | No | Yes | Yes | 6 | | |
| Waghmare, 2023 | Yes | Yes | Yes | Yes | Unclear | No | Yes | Yes | 6.5 | | |
| Zaldívar-Lopez, 2023 | Yes | Yes | Yes | Yes | No | No | Yes | Yes | 6 | | |
| AbdAllh, 2024 | Yes | Yes | Yes | Yes | Unclear | No | Yes | Yes | 6.5 | | |
| Almaw, 2024 | Yes | Yes | Yes | Yes | No | No | Yes | Yes | 6 | | |
| Obebe, 2024 | Yes | Yes | Yes | Yes | No | No | Yes | Yes | 6 | | |
| Zhuhua, 2024 | Yes | Yes | Yes | Yes | No | No | Yes | Yes | 6 | | |
| Lamine, 2025 | Yes | Yes | Yes | Yes | Unclear | No | Yes | Yes | 6.5 | | |
| Mulie, 2025 | Yes | Yes | Yes | Yes | No | No | Yes | Yes | 6 | | |
| Rashidifar, 2025 | Yes | Yes | Yes | Yes | Unclear | No | Yes | Yes | 6.5 | | |
| **Case-control** | | | | | | | | | | | |
| **Author Name/Year** | **Q1** | **Q2** | **Q3** | **Q4** | **Q5** | **Q6** | **Q7** | **Q8** | **Q9** | **Q10** | **Overall Quality** |
| Ukwah, 2017 | Yes | Yes | Yes | Yes | Yes | Unclear | No | Yes | Yes | Yes | 8 |
| Tay, 2017 | Yes | Yes | Yes | Yes | Yes | No | No | Yes | Yes | Yes | 7.5 |
| Sharma, 2017 | Yes | Yes | Yes | Yes | Yes | Unclear | No | Yes | Yes | Yes | 7.5 |
| Obateru, 2017 | Yes | Yes | Yes | Yes | Yes | No | No | Yes | Yes | Yes | 7.5 |
| Liyasu, 2017 | Yes | Yes | Yes | Yes | Yes | Unclear | No | Yes | Yes | Yes | 7.5 |
| Cerveja, 2017 | Yes | Yes | Yes | Yes | Yes | No | No | Yes | Yes | Yes | 7.5 |
| Casmo, 2018 | Yes | Yes | Yes | Yes | Yes | No | No | Yes | Yes | Yes | 7.5 |
| Udeh, 2019 | Yes | Yes | Yes | Yes | Yes | Unclear | No | Yes | Yes | Yes | 7.5 |
| Mbiandou, 2019 | Yes | Yes | Yes | Yes | Yes | Unclear | No | Yes | Yes | Yes | 8 |
| Abange, 2020 | Yes | Yes | Yes | Yes | Yes | No | No | Yes | Yes | Yes | 7.5 |
| Belay, 2020 | Yes | Yes | Yes | Yes | Yes | Unclear | No | Yes | Yes | Yes | 7.5 |
| Lengongo, 2020 | Yes | Yes | Yes | Yes | Yes | No | No | Yes | Yes | Yes | 7.5 |
| Zhao, 2022 | Yes | Yes | Yes | Yes | Yes | Unclear | No | Yes | Yes | Yes | 8 |
| Tanko, 2022 | Yes | Yes | Yes | Yes | Yes | No | No | Yes | Yes | Yes | 7.5 |
| Bejide, 2023 | Yes | Yes | Yes | Yes | Yes | No | No | Yes | Yes | Yes | 7.5 |
| Ishar, 2024 | Yes | Yes | Yes | Yes | Yes | No | No | Yes | Yes | Yes | 7.5 |
| Sarfo, 2024 | Yes | Yes | Yes | Yes | Yes | Unclear | No | Yes | Yes | Yes | 7.5 |
| **Cross-sectional questions:**  Q1. Were the criteria for inclusion in the sample clearly defined?  Q2. Were the study subjects and the setting described in detail??  Q3. Was exposure measured in a valid and reliable way?  Q4. Were objective, standard criteria used for measurement of the condition?  Q5. Were confounding factors identified?  Q6. Were strategies to deal with confounding factors stated?  Q7. Were the outcomes measured in a valid and reliable way?  Q8. Was appropriate statistical analysis used?  **Case – control design questions:**  Q1. Were the groups comparable other than the presence of disease in cases or the absence of disease in controls?  Q2. Were cases and controls matched appropriately?  Q3. Were the same criteria used for identification of cases and controls?  Q4. Was exposure measured in a standard, valid and reliable way?  Q5. Was exposure measured in the same way for cases and controls?  Q6. Were confounding factors identified?  Q7. Were strategies to deal with confounding factors stated?  Q8. Were outcomes assessed in a standard, valid and reliable way for cases and controls?  Q9. Was the exposure period of interest long enough to be meaningful?  Q10. Was appropriate statistical analysis used?  **Cohort design questions:**  Q1: Were the two groups similar and recruited from the same population?  Q2. Were the exposures measured similarly to assign people to both exposed and unexposed groups?  Q3. Was the exposure measured in a valid and reliable way?  Q4. Were confounding factors identified?  Q5. Were strategies to deal with confounding factors stated?  Q6. Were the groups/participants free of the outcome at the start of the study (or at the moment of exposure)?  Q7. Were the outcomes measured in a valid and reliable way?  Q8. Was the follow up time reported and sufficient to be long enough for outcomes to occur?  Q9. Was follow up complete, and if not, were the reasons to loss to follow up described and explored?  Q10. Were strategies to address incomplete follow up utilized?  Q11. Was appropriate statistical analysis used? | | | | | | | | | | | |
